# The Chloroplast Genome of *Lilium henrici*: Genome Structure and Comparative Analysis

**DOI:** 10.3390/molecules23061276

**Published:** 2018-05-26

**Authors:** Hai-Ying Liu, Yan Yu, Yi-Qi Deng, Juan Li, Zi-Xuan Huang, Song-Dong Zhou

**Affiliations:** Key Laboratory of Bio-Resource and Eco-Environment of Ministry of Education, College of Life Sciences, Sichuan University, Chengdu 610065, China; lhy921180@163.com (H.-Y.L.); yyu@scu.edu.cn (Y.Y.); yiqiden@gmail.com (Y.-Q.D.); lijuanxxn@163.com (J.L.); newshirley277@163.com (Z.-X.H.)

**Keywords:** Liliaceae, *Lilium henrici*, chloroplast genome, comparative analysis, phylogeny

## Abstract

*Lilium henrici* Franchet, which belongs to the family Liliaceae, is an endangered plant native to China. The wild populations of *L. henrici* have been largely reduced by habitat degradation or loss. In our study, we determined the whole chloroplast genome sequence for *L. henrici* and compared its structure with other *Lilium* (including *Nomocharis*) species. The chloroplast genome of *L. henrici* is a circular structure and 152,784 bp in length. The large single copy and small single copy is 82,429 bp and 17,533 bp in size, respectively, and the inverted repeats are 26,411 bp in size. The *L. henrici* chloroplast genome contains 116 different genes, including 78 protein coding genes, 30 tRNA genes, 4 rRNA genes, and 4 pseudogenes. There were 51 SSRs detected in the *L. henrici* chloroplast genome sequence. Genic comparison among *L. henrici* with other *Lilium* (including *Nomocharis*) chloroplast genomes shows that the sequence lengths and gene contents show little variation, the only differences being in three pseudogenes. Phylogenetic analysis revealed that *N. pardanthina* was a sister species to *L. henrici*. Overall, this study, providing *L. henrici* genomic resources and the comparative analysis of *Lilium* chloroplast genomes, will be beneficial for the evolutionary study and phylogenetic reconstruction of the genus *Lilium*, molecular barcoding in population genetics.

## 1. Introduction

The genus *Lilium* L., including about 115 species, is mainly distributed throughout cold and temperate regions of the Northern Hemisphere [1]. Many species of this genus have horticultural and food uses [2], especially their ornamental value. Lilies, the plants in the genus *Lilium*, has been widely known as one of the most important cut flowers in the world during the last 50 years [3]. Many *Lilium* species have large and beautiful flowers, with showy color and fragrant smell, and horticulturists produce many garden cultivars by interspecific hybridization according to their characteristics. China is rich in wild *Lilium* resources and possesses many endemic species. These rare *Lilium* species are a valuable raw material for hybrid breeding. In the traditional classification, species of *Lilium* have been divided into seven sections [4] using 15 morphological characters and many researchers have revised this classification. Currently, many molecular phylogenetic and morphological analysis inform our understating of this genus [5,6,7,8,9,10,11,12,13]. However, species-level relationships in *Lilium* are not yet clear [14] and more effective molecular markers are needed to determine interspecific phylogeny more accurately.

*Lilium henrici* Franchet, a *Lilium* species considered to be closely related to the *Nomocharis*, grows naturally in forests at an elevation of 2800 m in China’s Sichuan and Yunnan provinces [1]. It has a unique flower morphology and high horticultural value. However, the enormous economic value of *Lilium* plants for ornamental purposes, and as foods and medicines has led to excessive exploitation, causing their habitat to be fragmented [15,16], and unfortunately, many *Lilium* species, including *L. henrici*. were classified as endangered in the 2013 report and the impact of habitat degradation or loss has resulted in population decline. 

The typical chloroplast genomes of angiosperms usually encode 120 to 130 genes and are between 120 and 170 kb in length [17]. They are covalently closed molecules consisting of four parts, which are a large single copy (LSC) region, a small single copy (SSC) region, and a pair of inverted repeats (IRs) regions. As a consequence of conserved gene content and structure [18], the availability of plastome DNA sequences, an ability to resolve relationships at lower taxonomic levels [19], and maternal inheritance, the chloroplast genome has become particularly useful in phylogenetic and population genetic studies [20].

Advances in next generation sequencing technologies have revolutionized whole genome sequencing. The development of NGS methods achieve a breakthrough in sequencing technology and provide researchers with faster and cheaper methods to acquire chloroplast genome data [21]. More and more chloroplast genomic sequences are being analyzed, and phylogenetics have entered a new era at the same time. The chloroplast genome sequence of *L. henrici* obtained in this study can raise our awareness of this species, and the results will enrich the genetic information of the genus *Lilium* we already have, providing a theoretical basis for further study on the evolution of *Lilium* and species identification.

## 2. Materials and Methods

### 2.1. Plant Material, DNA Extraction and Sequencing

We sampled healthy and mature leaves of *L. henrici* from Danzha County (25°27′39′′ N, 98°15′27′′ E), Yunnan Province, China and preserved them in liquid nitrogen for further study. The corresponding voucher specimens were deposited at Sichuan University Herbarium (SZ Herbarium). Total genomic DNA of *L. henrici* was extracted from sampled leaves using the Plant Genomic DNA Kit (Tiangen Biotech, Beijing, China) following the manufacturer’s instructions. The isolated genomic was manufactured to average 350 bp paired-end(PE) library using Illumina Hiseq platform (Illumina, San Diego, CA, USA), and sequenced by an Illumina genome analyser (Hiseq PE150).

### 2.2. Chloroplast Genomic Assembly and Annotation

We used the software FastQC [22] v0.11.7 to evaluate the quality of sequenced raw reads. After quality assessment, we filtered the chloroplast genome related reads by mapping all the raw reads to the published chloroplast genome sequences in Liliaceae. All related reads were assembled to contigs using SOAPdenovo2 [23], and all contigs were sorted and joined into a single draft sequence with *L. taliense* as the reference in the software Geneious [24] v11.0.4. The gaps and ambiguous sequences were manually adjusted after Sanger sequencing. Annotations of chloroplast genome were conducted by the software Geneious v11.0.4, using other existent *Lilium* chloroplast genome sequences as references. Star/stop codons and intron/exon borders were edited manually after comparation with references. In addition, we used tRNAscan-SE [25] v2.0 to verify the identified tRNA genes. The circular map of *L. henrici* plastid genome was generated utilizing the OGDRAW program [26].

### 2.3. Simple Sequence Repeats (SSRs) Analysis

Perl script MISA [27] was used to detect chloroplast simple sequence repeats in twenty chloroplast genome sequences of *Lilium* (including *Nomocharis*). Its parameters were set as follow: the minimum numbers of repeats for mononucleotide, dinucleotides, trinucleotides, tetranucleotides, pentanucleotide and hexanucleotides were 10, 5, 4, 3, 3 and 3, respectively.

### 2.4. Analysis on Codon Usage

Codon usage of the *L. henrici* chloroplast genome was analyzed via codonW [28] software. Protein-coding genes (CDS) were filtered from *L. henrici* chloroplast genome with following conditions to reduce deviation of the results: (1) the length of every CDS must be more than 300 nucleotides [29,30]; (2) Repeat sequences were removed. Finally, 53 CDS in *L. henrici* were selected for further study (Table S1).

### 2.5. Genome Comparision

We downloaded *Lilium* and *Nomocharis pardanthina* [14,31,32,33,34,35,36,37,38,39] chloroplast genome sequences from GenBank and compared different chloroplast genomes in genus *Lilium*, including *L. superbum* (NC_026787), *L. longiflorum* (KC968977), *L. distichum* (NC_029937), *L. tsingtauense* (KU230438), *L. hansonii* (NC_027674), *L. cernuum* (NC_034840), *L. fargesii* (NC_033908), *L. taliense* (NC_034370), *L. bakerianum* (NC_035592), *L. brownie* (NC_035588), *L. duchartrei* (NC_035591), *L. henryi* (NC_035570), *L. lancifolium* (NC_035589), *L. leucanthum* (NC035590), *L. primulinum var. ochraceum* (KY748298), and *L. amabile* (NC_035988), *L. callosum* (NC_035989), *L. philadelphicum* (NC_035990). The mVISTA [40] program was used to compare *L. henrici* chloroplast genome sequence with other *Lilium* chloroplast genome sequences with Shuffle-LAGAN model, in which the annotation of *L. longiflorum* as the reference. We compared the borders between single copy regions (LSC and SSC) and inverted repeats (IR) regions among twenty *Lilium* (including *Nomocharis*) chloroplast genome sequences by using Geneious v11.0.4 software. 

### 2.6. Lilium Phylogenomic Reconstruction Based on Chloroplast Genome

Phylogenomic reconstruction of the genus *Lilium* was based on twenty-five whole chloroplast genome sequences, which were one species of *Cardiocrinum*, four species of *Fritillaria* and twenty species of *Lilium* (including *N. pardanthina*). All the sequences were aligned by MAFFT [41] and trimmed by trimAl [42]. Sequences, after alignment and trim, were used to select the best substitution model in the program jModelTest v2.1.7 [43] and the best model was GTR + I + G model. The phylogenomic relationship was inferred by maximum-likelihood method based on the GTR + I + G substitution model in MEGA 7.0 [44] with 1000 bootstrap replicates. Species in *Cardiocrinum* and *Fritillaria* were selected as outgroups.

## 3. Results and Discussion

### 3.1. Chloroplast Genome Features of the L. henrici 

**Figure 1:**
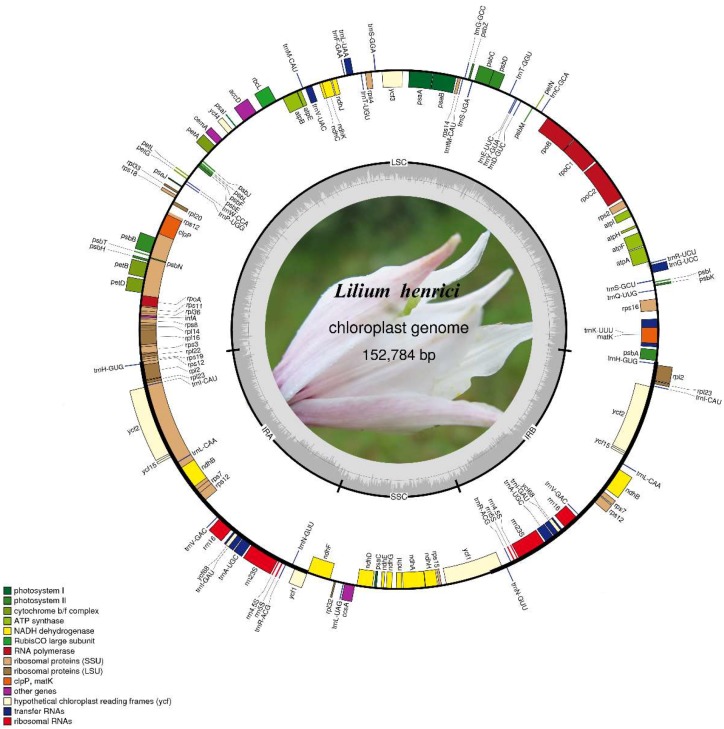
Gene map of the *Lilium henrici* chloroplast genome. The genes drawn outside and inside the outer circle transcribed clockwise and counter-clockwise, respectively. Genes of different functional groups are color coded. GC content and AT content are represented on the inner circle by darker gray and lighter gray, respectively.

We determined the *L. henrici* whole chloroplast genome sequence and deposited it to GenBank under accession number: MH136807. The complete plastid genome of *L. henrici* is a typical quadripartite structure with 152,784 bp in length, near to other *Lilium* chloroplast genome levels [14,31,32,33,34,35,36,37,38,39]. The genome, displaying a conserved structure found in most chloroplast genomes of plants [45,46], possesses an LSC region (82,429 bp), an SSC region (17,533 bp) and a pair of IR regions (26,411 bp) (Figure 1, Table 1). In the *L. henrici* plastid genome, the overall GC content is 37.0%. The GC content of the IR regions (42.5%) is higher than that of the overall genome (37.0%), LSC region (34.83%) and SSC region (30.59%). A total of 136 genes are detected, 20 of which are duplicated in the IR regions. There were 84 genes for protein-coding, 38 for tRNA, eight for rRNA and six for other functions among the 136 genes (Table 2). 

By containing several internal stop codons, the *infA*, *ycf1* (the shorter one), *ycf15*, and *ycf68* were interpreted as pseudogenes in our study. The same phenomenon also appears on other plants [47,48,49,50] or *infA* gene is lost in some taxa [51]. In a previous study, the *ycf1* and *ycf68* regions were considered to have potential function and required further study [47]. Different from other *Lilium* species, *L. henciri* is similar to *L. primulinum var. ochraceum* in having only has 23 bp duplicated. Other studies revealed only one *rps19* gene in *Lilium* close species, such as species in *Amana*, *Cardiocrinum* and *Fritillaria* [47,52,53,54]. Eighteen distinct genes (six of which are tRNAs) contain introns (Table S2), 3 (*rps12*, *clpP* and *ycf3*) of which contain two introns and the others only have one intron. The gene *rps12* is a trans splicing gene with its three exons located in the LSC and IR regions, respectively.

### 3.2. SSRs Analysis

**Figure 2:**
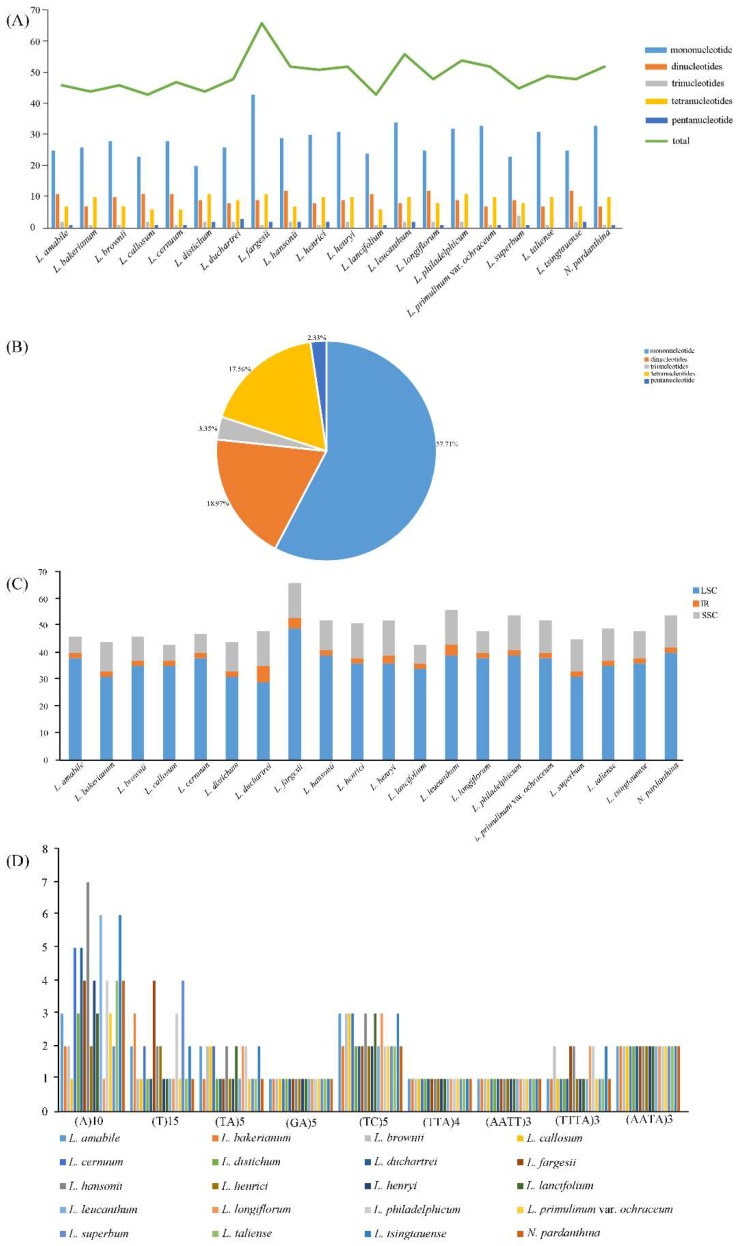
Analysis of simple sequence repeats (SSRs) in twenty *Lilium* (including *Nomocharis*) chloroplast genome sequences. (**A**) Number of different SSRs types detected in twenty *Lilium* (including *Nomocharis*) chloroplast genome sequences; (**B**) Presence of different SSRs types in all SSRs of twenty *Lilium* (including *Nomocharis*) chloroplast genome sequences; (**C**) Number of SSRs in the LSC, IR, SSC regions in twenty *Lilium* (including *Nomocharis*) chloroplast genome sequences; (**D**) Number of common SSRs in twenty *Lilium* (including *Nomocharis*) chloroplast genome sequences.

Simple sequence repeats (SSRs) usually composed of 1–6 nucleotide units [55], which have been accepted as important molecular markers for population variation studies [56,57,58]. We used MISA perl script to determine the SSRs loci in the twenty chloroplast genome sequences of *Lilium* (including *Nomocharis*) (Figure 2, Table S3). A total of 51 perfect microsatellites were detected in the *L. henrici* chloroplast genome (Figure 2A, Table S3). Moreover, 46, 44, 46, 43, 47, 44, 48, 66, 52, 52, 43, 56, 48, 54, 52, 45, 49, 48, and 52 SSRs were found in *L. amabile*, *L. bakerianum*, *L. brownie*, *L. callosum*, *L. cernuum*, *L. distichum*, *L. duchartrei*, *L. fargesii*, *L. hansonii*, *L. henryi*, *L. lancifolium*, *L. leucanthum*, *L. longiflorum*, *L. philadelphicum*, *L. primulinum var. ochraceum*, *L. superbum*, *L. taliense*, *L. tsingtauense*, and *N. pardanthina* (Figure 2A, Table S3). *L. fargesii* (66 SSRs) and *L. callosum* (43 SSRs), *L. lancifolium* (43 SSRs) possess the highest and lowest number of SSRs, respectively. All SSRs are divided into six types of microsatellites, which are mononucleotide, dinucleotide, trinucleotide, tetranucleotide, penta-nucleotide and hexanucleotide (Figure 2A, Figure 2B). The total number of mononucleotide repeats is more than the sum of the other types (Figure 2B) and all mononucleotide repeats consist of A or T bases. In the total SSRs loci, the repeats located in the LSC region is much higher than that in the SSC region and IR regions (Figure 2C). There are nine common SSRs detected in twenty *Lilium* (including *Nomocharis*) species (Figure 2D), which are (A)10, (T)15, (TA)5, (GA)5, (TC)5, (TTA)4, (AATT)3, (TTTA)3, and (AATA)3. Among the nine common SSRs, there are differences in the number and location of (A)10, (T)15, (TA)5, (TC)5 and (TTTA)3. These types of SSRs might be useful as molecular markers when studying *Lilium* genetic variability. 

### 3.3. Codon Usage Analysis 

We used 53 protein coding sequences from the *L. henrici* chloroplast genome for codon usage analysis. All protein coding sequences contain 21324 codons (Table 3). Leucine (2184 codons, approximately 10.24% of the total) and cysteine (241 codons, approximately 1.13% of the total) are the highest and lowest number of amino acids, respectively. Moreover, Met and Trp are encoded by only one codon. Except these two amino acids, others have obvious codon usage bias. For example, synonymous codons GCU, GCC, GCA and GCG encode alanine and the corresponding Relative Synonymous Codon Usage (RSCU) values [59] for these four codons in *L. henrici* are 1.75, 0.63, 1.16 and 0.47, respectively. There are 32 codons with a RSCU value more than 1. CDS GC content, about 37.4%, is similar with genome-wide GC content (37.0%). Codon usage bias of chloroplast genome may be affected by selection and mutation [60], and research on codon preferences can help us to better understand the exogenous gene expression and molecular evolution mechanisms of *L. henrici*.

### 3.4. Overall Sequence Variation of the Chloroplast Genomes among Species in Lilium

**Figure 3:**
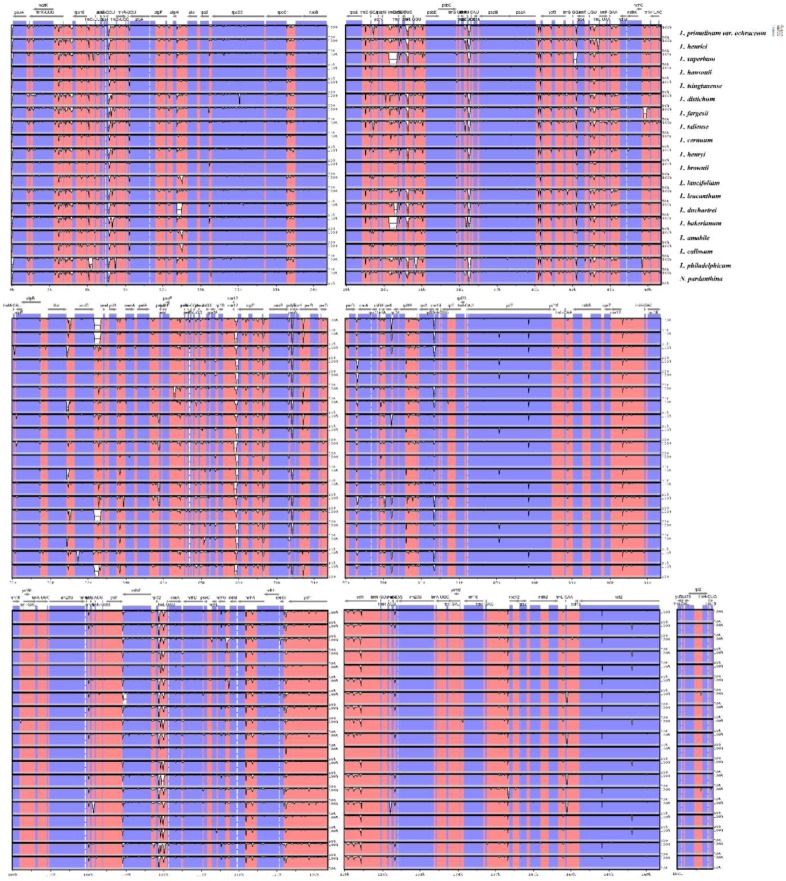
Sequence alignment of twenty *Lilium* (including *Nomocharis*) chloroplast genomes, with *L. longiflorum* as a reference. The y-axis indicates the percent identity between 50% and 100%. Genome regions colored represent protein coding regions, rRNA coding regions, tRNA coding regions or conserved noncoding sequences (CNS).

Preexisting whole plastome data in *Lilium* provides a basis for comparing genomic variation in the genus [61]. To compare the sequence variation within the genus, alignments among twenty *Lilium* (including *Nomocharis*) plastid genome sequences were implemented in the mVISTA program with Shuffle-LAGAN model (Figure 3). Overall, the comparative genomic analysis showed that twenty *Lilium* (including *Nomocharis*) chloroplast genomes were relatively conserved. Among the *Lilium* species, the IR region is more conserved than the LSC and SSC regions, similar with studies in other plants [47,51,61,62,63], and more variation were detected in the intergenic spacers in the LSC and SSC regions. In addition, fewer variations were found in protein coding regions. In the whole chloroplast genome sequences of *Lilium* species, some highly divergent regions including *matK*, *rpoC2*, *rps3*, *ycf2*, *ndhF*, *ndhH*, *trnK*-*rps16*, *trnS*-*trnG*, *atpH*-*atpI*, *trnT*-*psbD*, *trnF*-*ndhJ*, *accD*-*psaI* might be regarded as potential molecular markers for *Lilium* plants. Further work is needed to be implemented to verify whether these regions are suitable as molecular markers for phylogenetic studies of *Lilium*.

The main reason of change in the size of the chloroplast genome is the expansion/contraction of IR regions [64], and the location of the boundaries among the four chloroplast regions is useful for evolutionary studies [62]. The chloroplast genome organization is rather conserved within *Lilium*. The twenty *Lilium* (including *Nomocharis*) chloroplast genome sequences range from 151,655 bp (*L. bakerianum*) to 153,235 bp (*L. fargesii*), including an LSC region of 81,224–82,542 bp, an SSC region of 17,038–17,620 bp, and a pair of IR regions of 26,394–26,990 bp. The overall GC content of the complete genomes is 36.9–37.1%. There generally are the same gene order in all genomic sequences. We compared the borders of LSC, SSC and IR regions among twenty *Lilium* (including *Nomocharis*) plastid genomic sequences (Figure S1) and the IR regions of twenty *Lilium* (including *Nomocharis*) chloroplast genomes are conserved.

### 3.5. Phylogenetic Analysis

**Figure 4:**
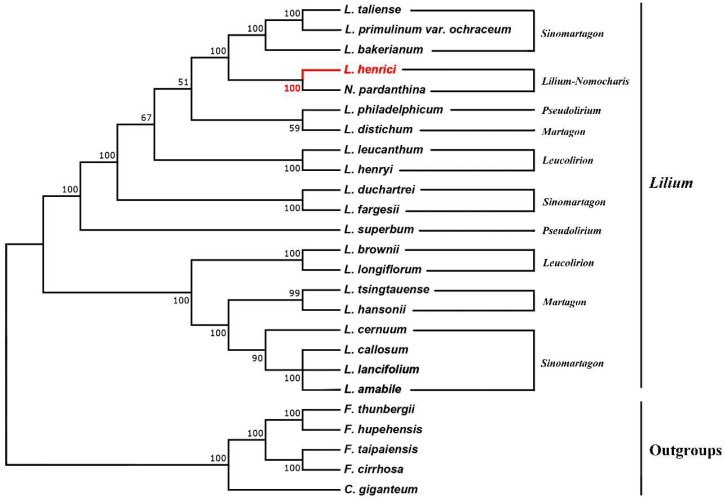
Molecular phylogenetic tree of the family Liliaceae based on the complete chloroplast genomes among 25 species. The tree was constructed using maximum likelihood (ML) algorithm and the GTR + I + G model.

In the previous reports, the chloroplast genomes are of great significance in the reconstruction of plants phylogenetic relationships and evolutionary history [63,64,65]. In our study, we constructed a phylogenetic tree using the sequences of the whole chloroplast genomes of twenty-five species in the family Liliaceae, including twenty *Lilium* (including *Nomocharis*) species and using five species in *Cardiocrinum* and *Fritillaria* as outgroups (Figure 4). All clades were strongly supported, and *L. henrici* was sister to *N. pardanthina*. The phylogenetic tree indicated that twenty species in *Lilium* (including *Nomocharis*) clustered into two groups. One group comprised section *Sinomartagon* (*L. cernuum*, *L. callosum*, *L. lancifolium* and *L. amabile*,), Section *Martagon* (*L. hansonii* and *L. tsingtauense*), and Section *Leucolirion* (*L. longiflorum* and *L. brownie*). Another group comprised Section *Sinomartagon* (*L. taliense*, *L. primulinum var. ochraceum* and *L. bakerianum*), *Lilium*-*Nomocharis* (*L. henrici* and *N. pardanthina*), Section *Pseudolirium* (*L. philadelphicum*), Section *Martagon* (*L. distichum*), Section *Leucolirion* (*L. leucanthum* and *L. henryi*), section *Sinomartagon* (*L. duchartrei and L. fargesii*), and Section *Pseudolirium* (*L. superbum*). 

The phylogenetic tree acquired in our study is consistent with previous studies [12,14,39]. The clade *Lilium*-*Nomocharis* was highly supported, and this conclusion was already proposed in many studies [9,12,66], and Gao et al. accommodated *Nomocharis* in *Lilium* [13]. The sister relationship between *L. henrici* and *N. pardanthina* is also supported by morphological and cytological evidence: (1) characteristic of inner perianth: sometimes anthocyanin rich [12]; (2) characteristics of karyotype: same basic chromosomal number (x = 12); karyotype asymmetry (3A type) [67]. However, we had used only a small number of species in *Lilium*-*Nomocharis* clade, other chloroplast genome sequences of species that might be included in this clade should be sequenced as soon as possible. 

## 4. Conclusions

We have sequenced the whole chloroplast genome of *L. henrici* using Illumina sequencing technology for the first time and compared its structure with other *Lilium* species. The plastid genome of *L. henrici* exhibits quadripartite structure. Compared with other chloroplast genomes in *Lilium*, it has similar size, genomic structure and gene order. And the chloroplast genome sequences in *Lilium* is relatively conserved at the boundaries of the IR regions and the SC regions. By comparing twenty *Lilium* (including *Nomocharis*) plastid genome sequences, we obtained 12 highly divergent regions. 51 SSRs were identified in *L. henrici.* We detected nine common SSRs in twenty *Lilium* (including *Nomocharis*) chloroplast genome sequences and these loci might play an important role in the further researches on the genetic structure of the genus *Lilium*. The research on codon usage of *L. henrici* shows that some amino acids have obvious codon usage bias and the codon preferences may help us understand the evolution mechanisms of *L. henrici* more deeply. Reconstructed molecular phylogenetic relationships using 25 complete chloroplast genome sequences in Liliaceae strongly supports the close sister relationship of *N. pardanthina* and *L. henrici*. We try to find morphological and cytological evidence to support their close relationship. Other whole chloroplast genome sequences of *Lilium*-*Nomocharis* clade need to be sequenced to understand the position of this clade or *Nomocharis* in the genus *Lilium*. The genome data obtained in this study will provide a theoretical basis for determinating the phylogenetic relationship of *Lilium*.

## Figures and Tables

**Table 1:** Genome features of *L. henrici* complete chloroplast genome.

Region	Chloroplast Features
Chloroplast genome size (bp)	152,784
LSC (bp)	82,429
SSC (bp)	17,533
IR (bp)	26,411
Total GC contents (%)	37.0
LSC GC contents (%)	34.83
SSC GC contents (%)	30.59
IR GC contents (%)	42.50
No. of total/unique genes	136/116
Total CDS length (bp)	113,441
Intergenic spacer (bp)	39,343
Protein-coding genes	78
tRNAs	30
rRNAs	4
Genes duplicated	20
Genes with intron(s)	18
Genes with a single intron	15
Genes with two introns	3
tRNAs with intron(s)	6

**Table 2:** List of genes present in the *L. henrici* chloroplast genome.

Classification of Genes		Name of Gene(s)	Number
RNA genes	Ribosomal RNAs	*rrn4.5*(x2), *rrn5*(x2), *rrn16*(x2), *rrn23*(x2)	8
	Transfer RNAs	*trnA-UGC*(x2), *trnC-GCA*, *trnD-GUC*, *trnE-UUC*, *trnF-GAA*, *trnfM-CAU*, *trnG-UCC*, *trnG-GCC*, *trnH-GUG*(x2), *trnI-CAU*(x2), *trnI-GAU*(x2), *trnK-UUU*, *trnL-CAA*(x2), *trnL-UAA*, *trnL-UAG*, *trnM-CAU*, *trnN-GUU*(x2), *trnP-UGG*, *trnQ-UUG*, *trnR-ACG*(x2), *trnR-UCU*, *trnS-GCU*, *trnS-GGA*, *trnS-UGA*, *trnT-GGU*, *trnT-UGU*, *trnV-GAC*(x2), *trnV-UAC*, *trnW-CCA*, *trnY-GUA*	38
Protein genes	Photosynthesis		
	Photosystem I	*psaA*, *psaB*, *psaC*, *psaI*, *psaJ*	5
	Photosystem II	*psbA*, *psbB*, *psbC*, *psbD*, *psbE*, *psbF*, *psbH*, *psbI*, *psbJ*, *psbK*, *psbL*, *psbM*, *psbN*, *psbT*, *psbZ*	15
	Cytochrome	*petA*, *petB*, *petD*, *petG*, *petL*, *petN*	6
	ATP synthase	*atpA*, *atpB*, *atpE*, *atpF*, *atpH*, *atpI*	6
	Rubisco	*rbcL*	1
	NADH dehydrogenease	*ndhA*, *ndhB*(x2), *ndhC*, *ndhD*, *ndhE*, *ndhF*, *ndhG*, *ndhH*, *ndhI*, *ndhJ*, *ndhK*	12
	ATP-dependent protease subunit P	*clpP*	1
	Chloroplast envelope membrane protein	*cemA*	1
Ribosomal proteins	large units	*rpl2*(x2), *rpl14*, *rpl16*, *rpl20*, *rpl22*, *rpl23*(x2), *rpl32*, *rpl33*, *rpl36*	11
	small units	*rps2*, *rps3, rps4*, *rps7*(x2), *rps8*, *rps11*, *rps12*(x2), *rps14*, *rps15*, *rps16*, *rps18*, *rps19*	14
Transcription/trnslation	RNA polymerase	*rpoA*, *rpoB*, *rpoC1*, *rpoC2*	4
	Miscellaneous proteins	*accD*, *ccsA*, *matK*	3
	Hypothetical proteins & Conserved reading frame	*ycf1*, *ycf2*(x2), *ycf3*, *ycf4*,	5
	Pseudogenes	*ycf15*(x2), *ycf68*(x2), *infA*, *ycf1*	6
Total			136

**Table 3:** Codon usage for *L. henrici* chloroplast genome.

Amino Acid	Codon	Number	RSCU	Amino Acid	Codon	Number	RSCU
Phe	UUU	794	1.33	Ser	UCU	446	1.66
	UUC	396	0.67		UCC	261	0.97
Leu	UUA	757	2.08		UCA	336	1.25
	UUG	421	1.16		UCG	142	0.53
	CUU	463	1.27	Pro	CCU	335	1.55
	CUC	137	0.38		CCC	190	0.88
	CUA	288	0.79		CCA	245	1.13
	CUG	118	0.32		CCG	95	0.44
Ile	AUU	893	1.42	Thr	ACU	434	1.62
	AUC	354	0.56		ACC	189	0.7
	AUA	638	1.02		ACA	343	1.28
Met	AUG	510	1		ACG	108	1.4
Val	GUU	439	1.5	Ala	GCU	506	1.75
	GUC	142	0.48		GCC	181	0.63
	GUA	435	1.48		GCA	335	1.16
	GUG	157	0.54		GCG	136	0.47
Tyr	UAU	686	1.64	Cys	UGU	181	1.5
	UAC	153	0.36		UGC	60	0.5
Ter	UAA	27	1.53	Ter	UGA	13	0.74
	UAG	13	0.74	Trp	UGG	383	1
His	CAU	408	1.59	Arg	CGU	284	1.37
	CAC	106	0.41		CGC	80	0.39
Gln	CAA	571	1.5		CGA	278	1.34
	CAG	190	0.5		CGG	103	0.5
Asn	AAU	828	1.57	Ser	AGU	348	1.29
	AAC	226	0.43		AGC	81	0.3
Lys	AAA	848	1.51	Arg	AGA	390	1.88
	AAG	273	0.49		AGG	110	0.53
Asp	GAU	692	1.6	Gly	GGU	466	1.27
	GAC	174	0.4		GGC	166	0.45
Glu	GAA	856	1.51		GGA	586	1.6
	GAG	275	0.49		GGG	245	0.67

## Supplementary Materials

The following are available online. Table S1: CDS used in the codon usage analysis and corresponding GC content, Table S2: Intron and exon information in the chloroplast genome of *L. henrici*, Table S3: Simple sequence repeats (SSRs) detected in the twenty chloroplast genome sequences of *Lilium* (including *Nomocharis*).
