# Supplementary material for: The Chloroplast Genome of Lilium henrici: Genome Structure and Comparative Analysis
Source: Molecules. 2018 May 26;23(6):1276. doi: 10.3390/molecules23061276 (PMC6100032; doi:10.3390/molecules23061276)
Supplement: Supplementary file 1 [file molecules-23-01276-s001.zip › Supplementary/Table S1 CDS used in the codon usage analysis and corresponding GC content.docx]

|  | | | | | |
| --- | --- | --- | --- | --- | --- |
| **Gene** | **Sequence Length**  **(bp)** | **GC content**  **(%)** | **Gene** | **Sequence Length**  **(bp)** | **GC content**  **(%)** |
| **accD** | 1470 | 32.8 | **psbB** | 1527 | 43.9 |
| **atpA** | 1524 | 40.2 | **psbC** | 1416 | 44.9 |
| **atpB** | 1497 | 42.4 | **psbD** | 1062 | 43.0 |
| **atpE** | 408 | 38.7 | **rbcL** | 1443 | 43.5 |
| **atpF** | 555 | 36.2 | **rpl2** | 819 | 43.7 |
| **atpI** | 744 | 37.2 | **rpl14** | 369 | 39.3 |
| **ccsA** | 966 | 32.1 | **rpl16** | 420 | 45.2 |
| **cemA** | 690 | 32.3 | **rpl20** | 348 | 35.6 |
| **clpP** | 615 | 41.6 | **rpl22** | 324 | 34.9 |
| **matK** | 1539 | 30.8 | **rpoA** | 999 | 35.6 |
| **ndhA** | 1092 | 34.1 | **rpoB** | 3207 | 38.4 |
| **ndhB** | 1566 | 37.9 | **rpoC1** | 2055 | 39.2 |
| **ndhC** | 363 | 37.2 | **rpoC2** | 4137 | 36.3 |
| **ndhD** | 1509 | 36.2 | **rps2** | 711 | 37.7 |
| **ndhE** | 303 | 31.4 | **rps3** | 657 | 35.0 |
| **ndhF** | 2229 | 31.7 | **rps4** | 606 | 36.8 |
| **ndhG** | 546 | 32.6 | **rps7** | 468 | 40.6 |
| **ndhH** | 1182 | 38.4 | **rps8** | 399 | 35.1 |
| **ndhI** | 540 | 33.1 | **rps11** | 417 | 45.8 |
| **ndhJ** | 477 | 38.2 | **rps12** | 372 | 41.4 |
| **ndhK** | 894 | 37.0 | **rps14** | 303 | 39.6 |
| **petA** | 963 | 39.1 | **rps18** | 306 | 35.3 |
| **petB** | 663 | 40.4 | **ycf1** | 5547 | 29.6 |
| **petD** | 504 | 38.1 | **ycf2** | 6633 | 37.1 |
| **psaA** | 2253 | 42.6 | **ycf3** | 513 | 39.6 |
| **psaB** | 2205 | 41.4 | **ycf4** | 555 | 39.3 |
| **psbA** | 1062 | 41.4 | **Total** | 63972 | 37.4 |
