# Supplementary material for: The Chloroplast Genome of Lilium henrici: Genome Structure and Comparative Analysis
Source: Molecules. 2018 May 26;23(6):1276. doi: 10.3390/molecules23061276 (PMC6100032; doi:10.3390/molecules23061276)
Supplement: Supplementary file 1 [file molecules-23-01276-s001.zip › Supplementary/Table S2 Intron and exon information in the chloroplast genome of L. henrici.docx]

| **Gene** | **Length (bp)** | | | | |
| --- | --- | --- | --- | --- | --- |
|  | **Exon 1** | **Intron 1** | **Exon 2** | **Intron 2** | **Exon 3** |
| **rps12** | 114 |  | 232 | 541 | 26 |
| **clpP** | 71 | 814 | 292 | 573 | 252 |
| **ycf3** | 126 | 717 | 228 | 722 | 159 |
| **atpF** | 144 | 784 | 411 |  |  |
| **ndhA** | 552 | 1049 | 540 |  |  |
| **ndhB** | 810 | 649 | 756 |  |  |
| **petB** | 6 | 731 | 657 |  |  |
| **petD** | 6 | 751 | 498 |  |  |
| **rpl16** | 9 | 989 | 411 |  |  |
| **rpl2** | 387 | 678 | 426 |  |  |
| **rpoC1** | 430 | 780 | 1625 |  |  |
| **rps16** | 40 | 887 | 212 |  |  |
| **trnA-UGC** | 38 | 810 | 35 |  |  |
| **trnG-UCC** | 24 | 682 | 48 |  |  |
| **trnI-GAU** | 37 | 937 | 35 |  |  |
| **trnK-UUU** | 37 | 2607 | 35 |  |  |
| **trnL-UAA** | 35 | 536 | 50 |  |  |
| **trnV-UAC** | 39 | 604 | 35 |  |  |
